# Supplementary material for: Biosynthesis of Vanillin by Rational Design of Enoyl-CoA Hydratase/Lyase
Source: Int J Mol Sci. 2023 Sep 4;24(17):13631. doi: 10.3390/ijms241713631 (PMC10487757; doi:10.3390/ijms241713631)
Supplement: Supplementary file 1 [file ijms-24-13631-s001.zip › ijms-2573446-supplementary.pdf]

International Journal of *Molecular Sciences*

MDPI

Supplementary Material

Biosynthesis of Vanillin by Rational Design of Enoyl-CoA Hydratase/Lyase

Qi Ye [1,†], Weizhuo Xu [2,†], Yanan He [1], Hao Li [1], Fan Zhao [1], Jinghai Zhang [1,*] and Yongbo Song [1,*]

[1] School of Life Sciences and Biopharmaceuticals, Shenyang Pharmaceutical University, 103 Wenhua Road, Shenhe District, Shenyang 110016, China
[2] School of Functional Food and Wine, Shenyang Pharmaceutical University, 103 Wenhua Road, Shenhe District, Shenyang 110016, China
* Correspondence: zhangjinghai@syphu.edu.cn (J.Z.); songyongbo@syphu.edu.cn (Y.S.); Tel.: +86-024-43520923 (Y.S.)
† These authors contributed equally to this work.

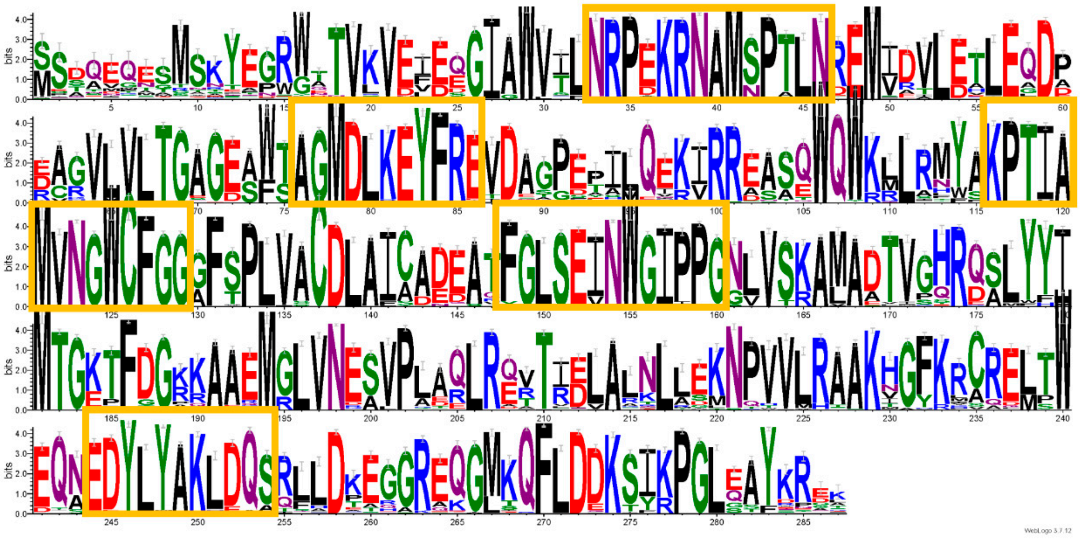

Figure S1. Conservation analysis of the enoyl-CoA hydratase/aldolase in sequence logo format. Major conserved regions are annotated with orange boxes. The overall height of the stack indicates the sequence conservation at that position, while the height of symbols within the stack indicates the relative frequency of each amino at that position.

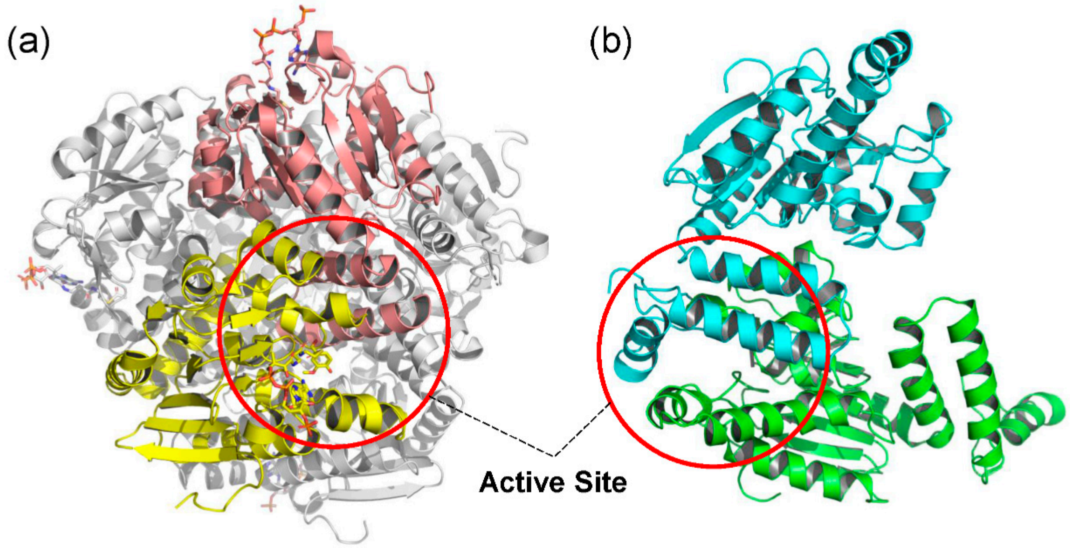

Figure S2. (a) X-ray structure of a native Hchl hexamer (PDB ID: 2VSS), (b) the homology model of Ech dimer. The common active site region of each structure is marked with red circle.

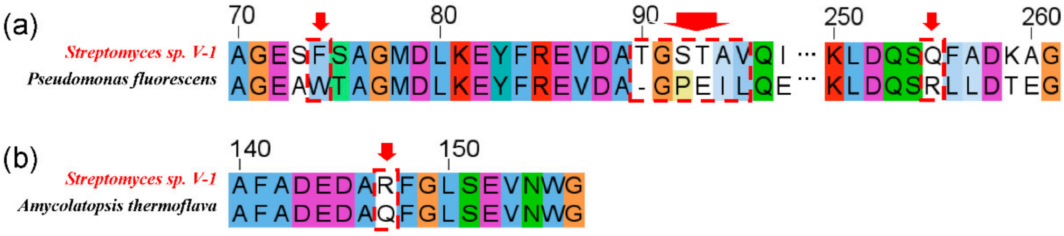

Figure S3. Partial sequence alignment of Ech from *Streptomyces* sp. V-1 with the homologous sequences from *Pseudomonas fluorescens* (a) and *Amycolatopsis thermoflava* (b).

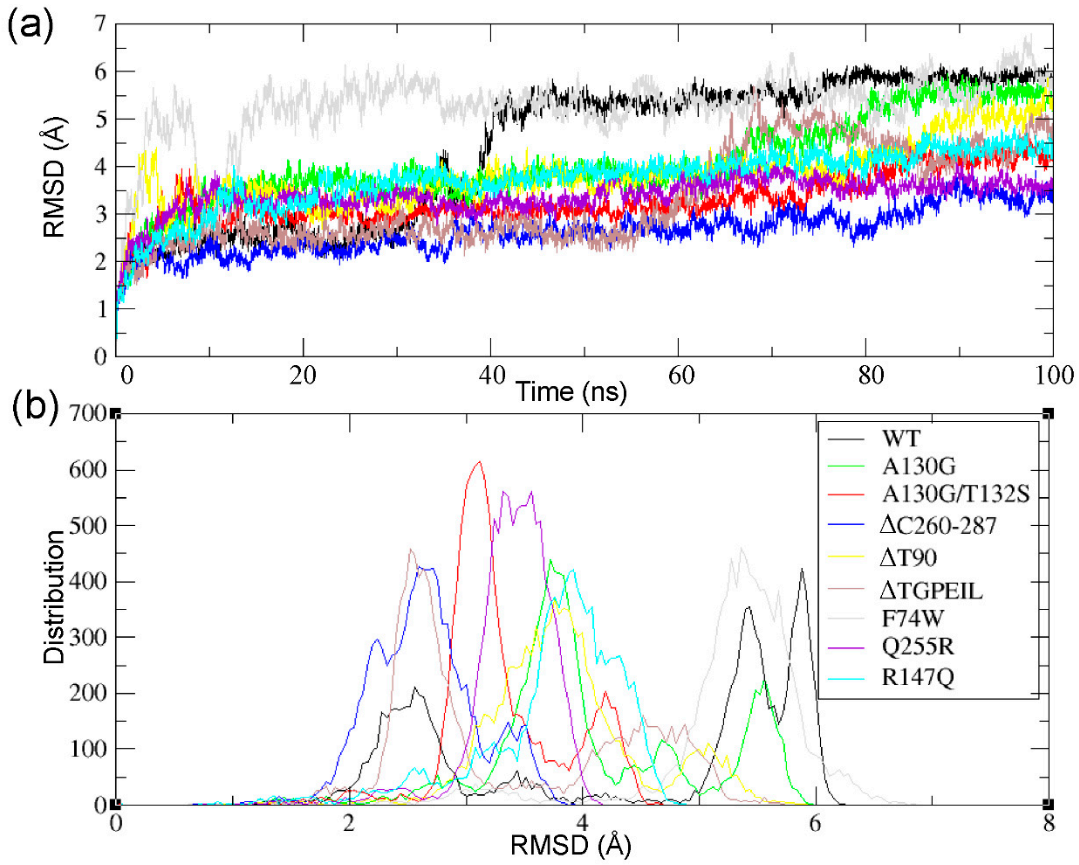

Figure S4. RMSD curves (a) and RMSD distribution (b) of WT and all mutants in MD simulations.

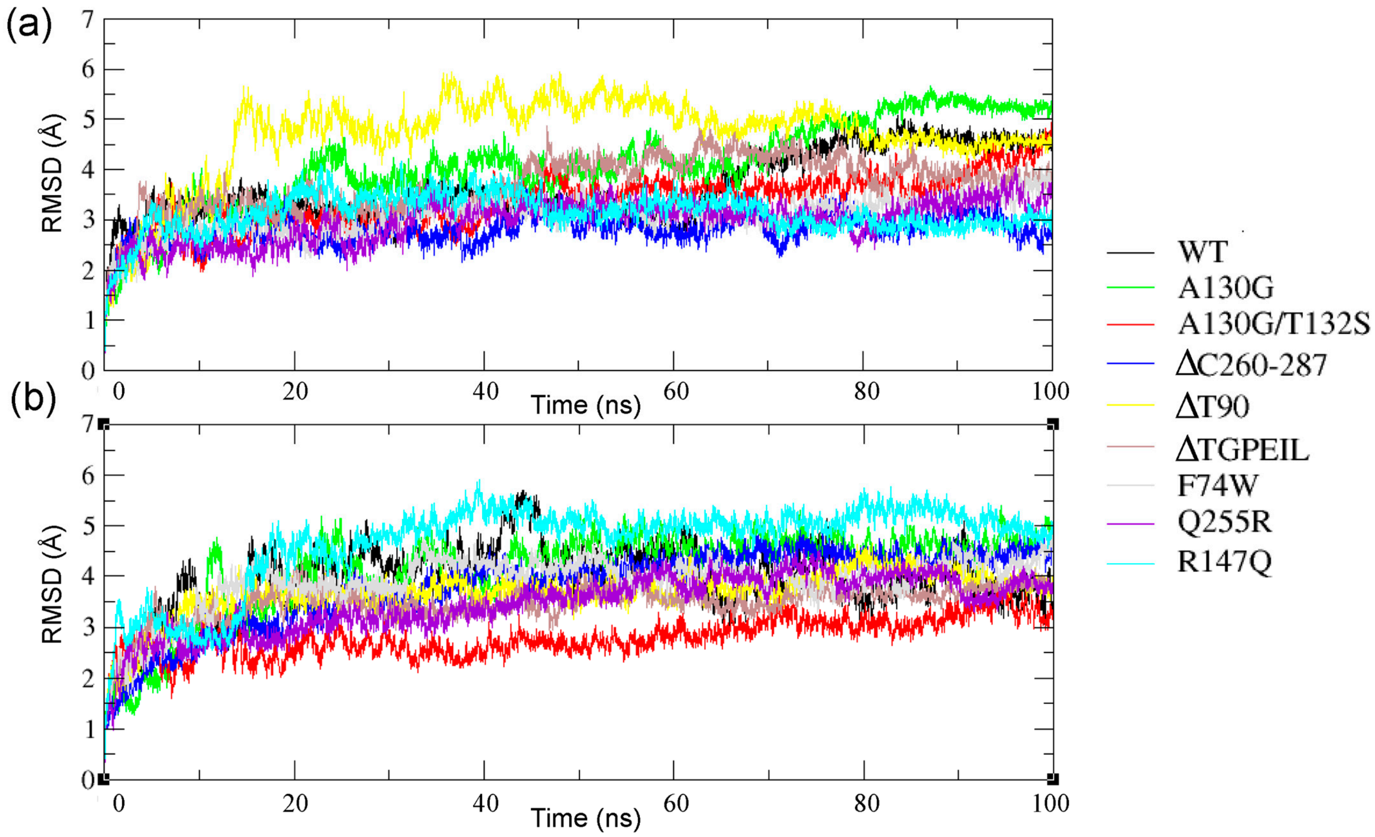

Figure S5. RMSD curves of WT and multiple mutants in the second (a) and third (b) MD simulations.

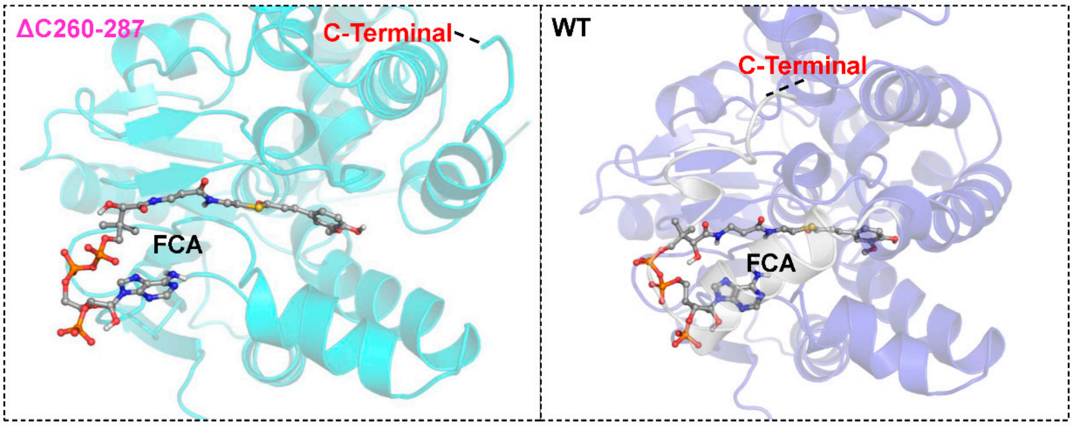

Figure S6. Structural effects of ΔC260-287 (cyan) compared to WT (blue) in MD simulations. Residues 260-287 in WT are shown as white cartoon and the C-terminal position is annotated.

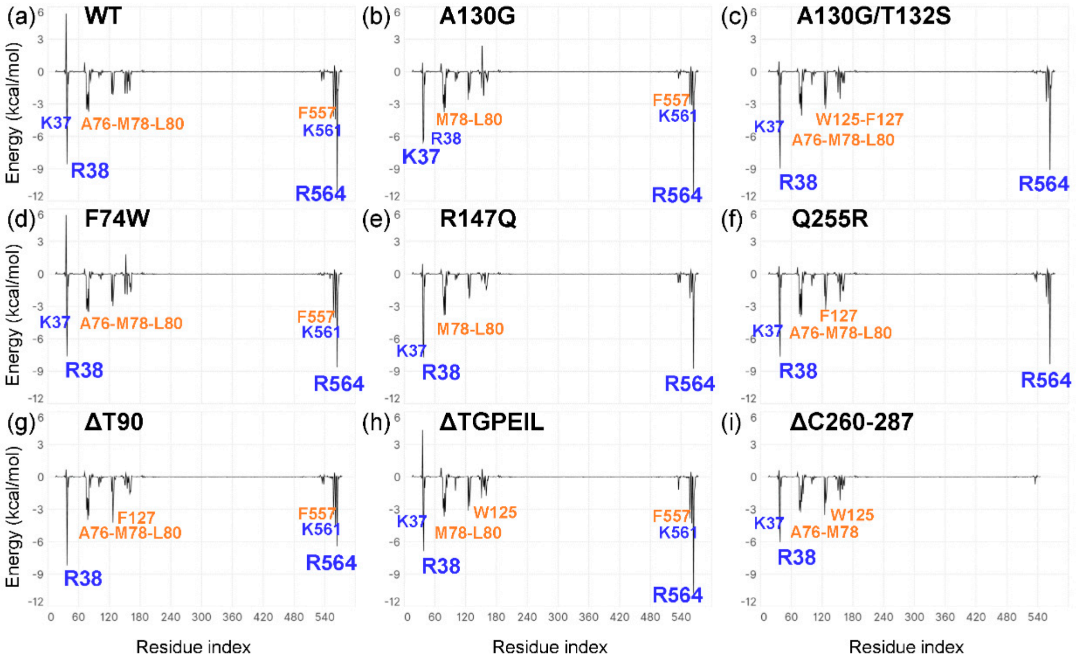

Figure S7. The per-residue energy decomposition of WT (a) and all mutants (b-i). The main basic (blue) and hydrophobic (organe) residues with high energy contribution are marked.

Table S1. Residues exhibit energy contributions to the binding free energy towards Ech and FCA

System	Residues in Chain A	Residues in Chain B
**WT**	**K37**, **R38**, A40, **A76**, G77, **M78,** D79, **L80**, W125, F127, G128, G129, S151, W155, G160	**F557**, L558, **K561**, **R564**, P565, G566, L567
**A130G**	**K37**, **R38**, A40, A76, G77, **M78**, D79, **L80**, W125, F127, G128, G129, S151, W155	**F557**, L558, **K561**, **R564**, P565, G566
**A130G/T132S**	**K37**, **R38**, A40, **A76**, G77, **M78**, D79, **L80**, R100, **W125**, **F127**, G128, G129, S151, W155, G160, V163	F557, K561, **R564**, P565, G566, L567
**F74W**	**K37**, **R38**, A40, **A76**, G77, **M78**, D79, **L80**, W125, F127, G128, G129, S151, W155, G160, V162, V163	**F557**, L558, **K561**, **R564**, P565, G566
**R147Q**	**K37**, **R38**, A40, A76, G77, **M78**, D79, **L80**, Y83, W125, F127, G128, G129, G160, G161, V162	F557, K561, **R564**, P565
**Q255R**	**K37**, **R38**, A40, **A76**, G77, **M78**, D79, **L80**, R100, W125, **F127**, G128, G129, W155, G160, G161, V162	F557, K561, **R564**
**ΔT90**	K37, **R38**, **A76**, G77, **M78**, D79, **L80**, W125, **F127**, G128, G129, W155, G160, G161, V162	**F557**, L558, **K561**, **R564**, P565
**ΔTGPEIL**	**K37**, **R38**, A40, A76, G77, **M78**, D79, **L80**, K81, Y83, R100, **W125**, F127, G128, G129, S151, I157, G160,	Y535, **F557**, L558, **K561**, **R564**, P565, G566
**ΔC260-287**	**K37**, **R38**, **A76**, G77, **M78**, L80, K81, Y83, **W125**, F127, G128, G129, S151, W155, P159, G160	-

Note: Energy contribution (ΔE) of all residues was less than -1 kcal/mol, where residues with high energy contribution (ΔE<-3 kcal/mol) are shown in bold.
